# Optoelectronic Capillary Sensors in Microfluidic and Point-of-Care Instrumentation

**DOI:** 10.3390/s100403771

**Published:** 2010-04-14

**Authors:** Michał Borecki, Michael L. Korwin-Pawlowski, Maria Beblowska, Jan Szmidt, Andrzej Jakubowski

**Affiliations:** 1 Institute of Microelectronics and Optoelectronics, Warsaw University of Technology, Koszykowa 75, 00-662 Warsaw, Poland; E-Mails: beblowska@imio.pw.edu.pl (M.B.); j.szmidt@imio.pw.edu.pl (J.S.); a.jakubowski@imio.pw.edu.pl (A.J.); 2 Département d’informatique et d’ingénierie, Université du Québec en Outaouais, 101 rue Saint-Jean-Bosco, Gatineau, QC J8X 3X7, Canada; E-Mail: michael.korwin-pawlowski@uqo.ca

**Keywords:** fiber-optic sensors, photonic sensors, optical capillaries, optical fibers, chemical sensors, optochemical sensors, capillary sensors, liquid-core sensors, hollow-core sensors, biochemical sensors

## Abstract

This paper presents a review, based on the published literature and on the authors’ own research, of the current state of the art of fiber-optic capillary sensors and related instrumentation as well as their applications, with special emphasis on point-of-care chemical and biochemical sensors, systematizing the various types of sensors from the point of view of the principles of their construction and operation. Unlike classical fiber-optic sensors which rely on changes in light propagation inside the fiber as affected by outside conditions, optical capillary sensors rely on changes of light transmission in capillaries filled with the analyzed liquid, which opens the possibility of interesting new applications, while raising specific issues relating to the construction, materials and instrumentation of those sensors.

## 1. Introduction

Chemical and biochemical sensors using optical fibers as sensing elements are the subject of reviews and books, of which notable are the biennial reviews of Wolfbeis [1–4] and among the recent book publications, those by Ringhini, Tajani and Cutolo [5] and by Baldini, Chester, Homola and Martellucci [6]. A distinct sub-class is sensors utilizing fiber optic capillaries and optical waveguides as sensing heads for chemical and biochemical applications. Among the reviews covering this type of sensors are [7,8] as well as more recent reviews of Baldini and Giannetti [9].

Perhaps the most ambitious project involving fiber optic capillary sensors was the European Union IST-FP6 Integrated Project CLINICIP [10]. The project involved in the 2004–2007 period 15 European institutions and had as its main objective the development of a low-risk monitoring and control system which would allow maintaining metabolic control of critically ill patients in an intensive care unit. The system monitored patient blood glucose and also allowed monitoring of O_2_, pH and CO_2_ levels. Three types of sensors were developed and evaluated in the course of the project, based on amperometric, infrared spectrometric and fluorometric fiber optic principles. Although in the final instance the latter was terminated in the project due its unsatisfactory performance as glucose sensor, it had been further developed as a competing technology and back-up system [10].

In this paper we present a review of fiber optic capillary sensors (FOCap), trying to classify them primarily from the standpoint of construction and instrumentation, rather than from the standpoint of applications. Capillary sensors can be considered a sub-set of liquid-core sensors or hollow-core sensors, examples of which are provided [11–20]. Much attention was been drawn to the two latter sub-classes in their advanced form of photonic bandgap fibers or microstructured optical fibers. Those devices often are built to perform similar sensing functions and may be used in similar kinds of applications as the FOCaps. We will be focusing on the simple instruments using short sections of FOCaps as sensing elements, not merely as conduits of fluids.

There are many advanced optoelectronic and fiber-optic sensors for examining liquids whose operation is based on precise control of the volumes and movement of liquids within the sensing head. This control is possible when capillary fibers are being used, as is the case in electrophoresis systems and liquid capillary waveguide cells. A very important step toward increased use of microfluidic sensing is reduction of the total sample volume, as distinct from the volume under examination. This distinction can be better explained if we take the case of a micropump used to introduce a sample into a sensor head. One can have small sample volume under examination even though relatively large volumes of the sampled liquid have to be drawn, and are wasted. When the liquid wets the capillary walls, the volume of the sample can be reduced because the capillary can perform multiple functions: it can serve as a micropump, as a sample holder and as the head of the optoelectronic sensor. Another problem encountered in many biological applications, especially in point-of-care applications, is the need for one-time utilization of the sensing capillary. The cost of single-use sensors can be reduced by dividing the sensor head into a disposable capillary optrode and a durable head base–but this introduces the problem of repeated coupling of the capillary with the optoelectronic leads of the system. The last important problem in microfluidic sampling is identification. This can be done by using single- or multi-parametric processing. Single-parameter processing employs selective sensing layers of chemical or biological transducers. Layers that are chemically sensitive may have many components which often include pH optical indicator dyes, while biologically sensitive layers consist of antibody or microorganisms immobilized in the film material. Multi-parameter processing uses the information obtained from a sample as it is subjected to external stimulus such as local heating during the measuring cycle. Application of heating can cause many reactions, for example: a phase change from liquid to gas and back again to liquid, or a change of measurable sample parameters such as the index of refraction or light-scattering properties. The information collected during the measuring cycle has an indirect relation with the investigated sample parameters and can be used for sample classification by an artificial neural network (ANN).

The best known capillary sensors use the principle of indirect measurement, with the liquid acting as a transducer of temperature or pressure into visible information [8]. For example, an alcohol thermometer has two important elements: the sensor head and the elements that enable conversion of the information from the thermal expansion of a liquid into a temperature reading. The sensor head is a bulb connected with a capillary. The converting elements can consist of optoelectronic circuits. When the temperature can be independently measured and controlled, the changes induced in the liquid by a change of temperature can be used to sense thermal expansion of the liquid. This thermal expansion is affected by the atmospheric pressure outside the liquid and by the capillary action. When at least one of the capillary ends is open, the liquid flow can be examined. The flow of liquid through the capillary is affected by its viscosity [21]. When the temperature of the sample filling the capillary crosses the boiling point of that particular liquid, the flow is non-uniform because a bubble is formed inside the capillary. If the capillary is illuminated, this bubble can induce light switching (LS). Temperature changes can also induce changes in light scattering in the examined sample and in the sample’s index of light refraction that can be detected by optoelectronic circuits [22]. The sensitivity to refractive index changes as found by measuring the wavelength shift of whispering-gallery-mode resonance is greater than in other microcavities [23]. The information collected can be converted into a liquid finger-print that can then be used in classifying samples of liquids examined by means of fiber-optic capillary (FOCap) sensors.

**Figure 1.**
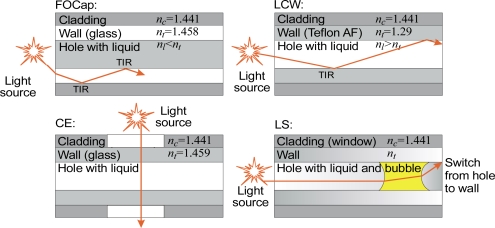
Examples of optical signal path in capillary heads.

The index of refraction and the light-scattering properties of a liquid sample depend on its composition. Information about sample composition can be processed with high accuracy in liquid capillary waveguide (LCW) cells connected to an optical spectroscope. Capillaries can be also used for liquid composition separation which is the principle of capillary electrophoresis (CE). Examples of possible optical configurations of capillary heads and variant light paths are presented in Figure 1. Other constructions of optical capillary sensors are also possible, for example, using liquid-core optical ring resonators [24].

## 2. Laboratory Microfluidic Systems

Microfluidic systems in such forms as labs-on-chips (LoCs), micro-total-analysis systems (μTas), and liquid chromatography (LC) or capillary electrophoresis (CE) and optical coherence tomography systems (OCT) are currently used in the laboratory environment. Such systems are both complex and costly [25]. Work is underway to develop a fully integrated chip-scale optical sensing system. One of the first steps in this direction was the active thin-film compound semiconductor optical device integrated with a digital microfluidic platform presented in [26].

### 2.1. Precise control of Fluid Volumes and Movement in the Sensing Heads

**Figure 2.**
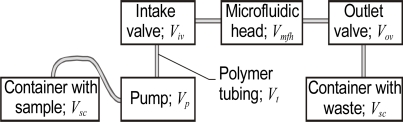
The setup for control of the volume and movement of liquid.

Small fluid volumes of micro- and nano-liters must be precisely controlled in any microfluidic system or sensor. The setup commonly used for liquid volume and movement control is presented in Figure 2. LoCs and μTas may have integrated pumps [27].

Such a setup requires samples with a relatively large volume *V_s_* which is the sum of the volumes contained in the various components as illustrated in Figure 2:

(1) $$V_{s}=V_{p}+V_{\mathrm{iv}}+V_{\mathrm{mfh}}+V_{\mathrm{ov}}+V_{t}$$

Since the examined sample volume *V_es_* is part of the volume in the microfluidic head, it follows that:

(2) $$V_{\mathrm{es}}\leq V_{\mathrm{mfh}}$$

This type of setup can be used during the analysis or design of microfluidic devices or when the volume to be sampled is extensive, for example, when river, lake or sea water is being monitored. Systems like this can be assembled relatively quickly in laboratory conditions since their major components–micropumps [28], microvalves [29] with integrated capillaries for connecting purposes, connectors and polymer microtubing [30]–are commercially available at reasonable prices. For example, a micropump without driver currently costs about €20.

### 2.2. Microfluidic Systems with LCW Cells

**Figure 3.**
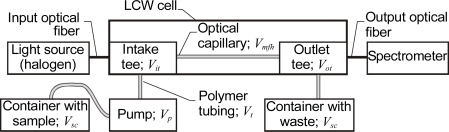
Microfluidic system with an LCW cell.

The microfluidic system presented in Figure 3 uses an LCW cell as the test head and tee connectors as the valves connecting the optical fibers and the tubing. LCW cells are commercially available at World Precision Instruments, Inc. Typical capillaries used in such cells have optical path lengths from 2 to 500 cm, inner diameters of 550 μm and internal volumes from 5 to 1,250 μL. Both continuous and discrete introduction of samples into the LCW cell are possible.

When a syringe is used to introduce a sample, the discrete sample volume *V_s_* should be [31]:

(3) $$V_{s}\geq 3\cdot (V_{t}+V_{\mathrm{it}}+V_{\mathrm{mfh}}+V_{\mathrm{ot}})\approx 3\cdot V_{\mathrm{es}}$$

The optical capillary in the LCW can be made of Teflon AF2400 or of fused silica tubing coated inside with a low-refractive-index polymer. The refractive index of the tubing must be lower than that of the examined liquid. For example: water at 20 °C has an index of refraction of 1.33; acetone, 1.36; and ethyl alcohol, 1.36; while Teflon AF2400 for wavelengths greater than 380 nm has a refractive index below 1.29 [32]. This ensures that light from the input optical fiber will be guided through the liquid in the capillary to the output fiber connected with the spectrometer. LCW systems can be used to extend the sensitivity of conventional absorbance spectroscopy by two or more orders of magnitude. They can also eliminate the need for sample preconcentration in some analyses. For example, the iron Fe(II) concentration above 0.2 nmol·dm^−3^ in natural waters can be directly examined by monitoring with light at a wavelength of 562 nm [33].

Hollow waveguides were proposed for detection of gases and liquids [34–41]. Sections of small bore (i.d. of 530 μm, 700 μm and 1,000 μm) silica capillaries of 20–25 cm length coated on the inside walls with silver and silver halide were used as sensors of CO_2_ in N_2_ in the range from 200 ppm to 10,000 ppm, showing linear response to the CO_2_ concentration [34].

Silica capillaries of four meter length and 700 μm i.d. with the inner wall surface coated with a reflecting layer of Ag/AgI showed excellent light transmission in mid-infrared range with an attenuation of 0.1 dB/m at 10.6 μm and functioned both as optical waveguides and gas cells. They were used for gas detection in conjunction with SiC globar light sources and FTIR spectrometers down to ranges of ppb for chlorinated hydrocarbons and ppm for 1,4 dioxane [35]. In conjunction with a supported capillary membrane those capillaries were used to detect the concentration of volatile organic compounds in water down to ppm level [36]. The same kind of hollow waveguides detecting the attenuation of room-temperature quantum cascade lasers InGaAs/AlInAs/InP at 971 cm^−1^ sensed ethyl chloride in air down to concentrations of 0.5 ppm (v/v) [37]. Hollow waveguides were used with quantum cascade laser spectrometer as an online microliter sensor for gas chromatography [38]. Another version of hollow waveguides used polyethylene capillaries with silver reflective layers additionally covered with hydrophobic films to attract and detect organic compounds dissolved in water with sensitivity down to 50 ppm v/v using conventional FTIR spectrometry [39]. Further improvements allowed reducing the volumes of the samples to 50 μL and sensitivities to 10 to 100 ppm, depending on the compound [40,41].

A distinct advantage of the waveguiding properties of the capillary is the capability to integrate the signal over an increased surface area without simultaneously increasing the background noise from the detector. A biosensor was demonstrated to achieve limits of two orders of magnitude greater sensitivity than was achieved using the same immunoassay reagents in a fiber optic biosensor or a planar array biosensor [42].

Some proposed sensing applications of hollow capillaries are removed from the classical sensing of gas and liquids. Branzalov et al. proposed a fiberoptic displacement sensor with a short hollow metal optical waveguide [43]. Filling silica/Ag/AgI coated hollow waveguides with silver nanoparticles, thus combining capillary sensing with nanotechnology, Shi et al. have developed a molecular sensor based on double substrate surface enhanced Raman scattering with greatly improved sensitivity for molecular detection. In tests of detecting R6M molecules in water at 10^−6^ M concentrations those sensors showed sensitivities 100 times better than in direct sampling [44].

### 2.3. CE Systems

Capillary electrophoresis is used for precise analysis of liquids containing ions that can be separated in an applied electric field. A large body of literature exists on this topic, but its review is beyond the scope of this paper. However, since CE represents a major application of capillaries in what can be considered a form of sensing, it is mentioned here, with references to recent specialized review articles [45–49].

**Figure 4.**
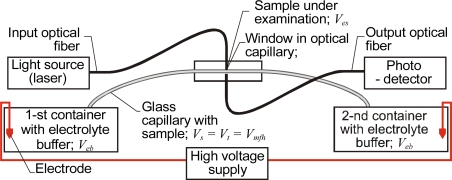
CE system using fiber optics.

Briefly, in CE the electrically charged ions of the analyte move from one container to another under the influence of an electric field. The high voltage supply acts as a pump and causes separation of ions by their charge, frictional forces and mass. The time-dependent concentration of separated ions can then be measured by optical detection devices [50]. The most common systems are based on detection of optical absorbance or on fluorescence analysis across the capillary, as shown in Figure 4. Most often CE capillaries are made of fused silica, but polymer capillaries have also been proposed to take advantage of their lower index of refraction [51]. The separation of ions improves when the capillary’s inner diameter is small, but for the purposes of optical detection, a larger inner diameter gives better sensitivity. Both effects have technical limits, so in practice the capillary inner diameters in CE range from 25 μm to 75 μm. The focusing of light on the capillary is thus an important design consideration. The sample volume under examination in CE is very small and can be approximated as the width of the light beam crossing the capillary multiplied by the area of the capillary’s cross-section. Decreasing the sample volume *V_s_* can be achieved by the on-line pre-concentration method, in which the examined sample is injected directly into the capillary [52]. The volume of the CE sample is then comparable to that of an LCW sample. The combination of CE and laser-induced fluorescence (LIF) represents the current state of the art in terms of sensitivity for microcolumn separation techniques. In particular, LIF detection coupled with capillary electrophoresis (CE) has achieved detection limits approaching the single molecule level, but only for molecules that have fluorescent properties [53]. The disadvantages of CE are the need for conditioning of new capillaries and the requirement to regenerate the inner walls of the capillaries between the measurements [54].

### 2.4. OCT Systems

Optical coherence tomography is an interferometric imaging technique performed using fiber-optic Michelson interferometer with low-coherence-length light source. The fiber-optic implementation provides a compact system that can be interfaced to a variety of clinical imaging instruments. One interferometer arm contains a probe that focuses, scans the light onto the sample, and collects the backscattered light. The second interferometer arm is a reference path with a translating mirror or a scanning delay line. The interference fingers are detected and demodulated to produce the measurement of the magnitude and echo delay time of light backscattered from the samples [55]. The outputs of OCT systems are three-dimensional images which resolutions that depend on the properties of the light source - superluminescent diodes and ultrashort pulsed lasers [56].

**Figure 5.**
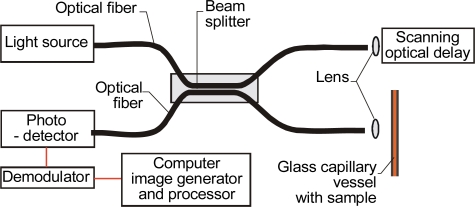
OCT system using optical capillary as a vessel.

OCT systems use near-infrared light for acquisition and processing of optical signals from optically scattering media. The relatively long wavelength light penetrates into the scattering medium [57,58]. Commercially available OCT systems have found diverse applications, including art conservation and diagnostic medicine, notably in ophthalmology where they can be used to obtain detailed images from within the retina. The tomography image can be acquired from a capillary that is used as a liquid vessel in the setup presented in Figure 5.

Work [59] presents a method of determination of optical parameters of blood such as the index of refraction, the attenuation coefficient, and the scattering anisotropy factor, measured at 1,300 nm. The estimations of the attenuation coefficient and of the anisotropy factor are based on a numerical algorithm fit to an experimentally acquired optical coherence tomography (OCT) heterodyne efficiency curve.

## 3. Microfluidic Sensors

Even more than for laboratory situations, microfluidic sensors for industrial and field applications have to be inexpensive to purchase and operate as well as simple to use, and the choice of components must take these requirements into account. As a light source, light emitting diodes (LEDs) are preferable to laboratory laser light sources. However, with LEDs, the optical signal to the detection unit is one or two orders of magnitude lower, which means that background light has to be eliminated from the measured signal. This can be done with light modulation on the source side and signal filtering in the photoreceiver. The receiver can be built with an integrated amplifier-photodetector with a filter, such as the monolithic photodiode and single-supply transimpedance amplifier OPT101 in conjunction with the universal active filter UAF42, all available from Texas Instruments [60,61]. The use of LEDs also limits the optical sensor detection system to the measurement of light intensity. Halogen or deuterium spectrometer lamp sources, even in original equipment manufacturer (OEM) versions, are too costly for most sensors. In their place, a few selected and switched LEDs can be used [62]. The components of a microfluidics sensor also have to be replaceable or at least easily renewable by a non-professional user.

In addition to simple, inexpensive components, microfluidic sensors must have appropriate sampling arrangements. Precision microsyringes have been proposed for filling capillaries with bio-reactive mixtures [63], but they can only be used by a trained technician. Liquids can be sampled directly using optical capillaries, but the reproducibility and effectiveness of coupling light into and out of the capillary have to be assured [64].

### 3.1. Capillary Absorbance Sensors with Sensitive Layers

**Figure 6.**
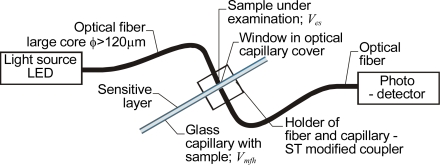
Optical capillary sensor with optical fibers for absorption measurements intended for monitoring transfluent samples.

The basic setup for a capillary absorbance sensor with a sensitive layer intended for monitoring transfluent samples is shown in Figure 6.

This device was proposed and evaluated as a carbon dioxide sensor by Weigl and Wolfbeis [8]. As a light source, an LED with a wavelength of 590 nm was used. The intensity signal was monitored on a Hewlett-Packard 8153A lightwave multimeter. To facilitate light coupling, optical fibers with 125 μm diameter cores were selected. The fibers were positioned in an ST coupler with a hole for the capillary drilled in the center. The capillary was permanently mounted and its inner walls were coated with chemically sensitive layers. In the microfluidic channel, the capillary was connected by polyethylene tubing to a container holding the sample solution–a mixture of buffer liquid and carbon dioxide gas. This capillary sensor performed better than a membrane sensor.

Several attractive features were listed for capillary waveguide optrodes [64]: (a) they can serve as sample cavities or flow cells for gases and liquids; (b) they are suitable for direct sampling; (c) their relative signal change can be optimized by adjusting the optical pathlength; (d) the optical pathlength is much longer compared to other optrode designs based on absorption or reflection, therefore the coatings can be made thinner, and less loaded with chemically active components; (e) the sensors are hardly cross-sensitive to color and turbidity of the sample; (f) cheap light sources and detectors can be used in connection with these novel capillary waveguide optrodes.

### 3.2. Optical Evanescent Field Sensors with Capillary Optrodes

**Figure 7.**
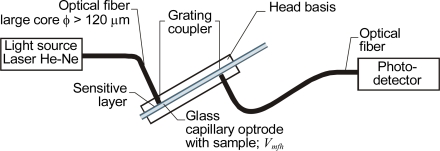
Optical capillary evanescent field sensor with sensitive layer intended for monitoring transfluent samples.

One of the first designs for an optical capillary evanescent field sensor, where the capillary functioned as an optrode, was proposed by Weigl and Wolfbeis in [8]. The sensor head used the same sensitive layer as the head shown in Figure 5 and was also intended for monitoring transfluent samples, but it was constructed differently. Light was coupled into the walls of the glass capillary with a grating made on the wall. The light then propagated in the capillary’s walls. The capillary was placed on a holder plate where the light source and photodetector positions were set at right angles to the capillary axis, for example, by means of an optical fiber. This construction was more suitable for integration because it allowed for an optical coupling that was not permanently fixed. To improve the coupling efficiency, a He-Ne laser was used as a source (Figure 7).

**Figure 8.**
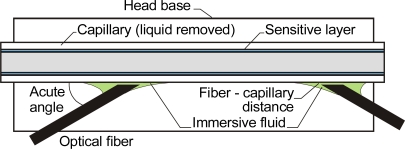
Capillary evanescent field head with optical fibers positioned at an acute angle [66].

In a different design proposed by Paprocki *et al.* [65], the propagating light has an evanescent field component. This penetrates the sensitive layer, which is about 2 μm thick, changing the real and complex part of the coefficient of light refraction in the presence of carbon dioxide. The result is a longer optical path and greater sensitivity than for the sensor in Figure 5. The main drawback of this construction is its use of a He-Ne laser. A method of overcoming the low efficiency of coupling to the capillary walls was presented in [66]. Instead of gratings, this head had the optical fiber and immersive liquids positioned at an acute angle to the capillary axis (Figure 8).

The fibers were positioned on the base of the sensor head. For each sensing procedure, the capillary was placed on the base, and immersive fluid drops were then deposited. In order for the fluid to make contact, both fibers and capillary had to be made from materials with similar surface tensions. This optrode was proposed for analyzing the carbon dioxide content of liquids, as were the sensors shown in Figures 5 and 6, but the sensing here did not employ a transfluent mode. So as not to disturb the position of the capillary the liquid was introduced with a syringe and held in place until the chemical reaction with the sensitive layer occurred. Then the liquid was pumped out, and the capillary was dried and replaced on the base. The sensor performed as quickly and accurately as the previously mentioned carbon dioxide sensors and had the cost advantage of using an LED light source and replaceable optrodes, but the measurement procedure requires a trained user.

### 3.3. Optical Capillary Sensors with Fluorescent, Sensitive and Light-Scattering Layers

**Figure 9.**
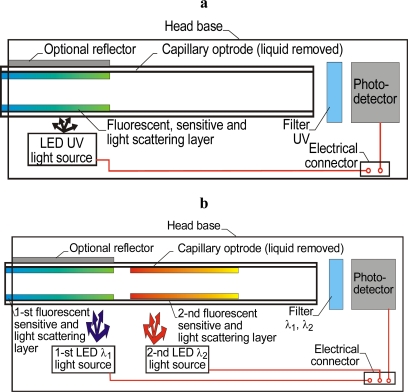
**a)** Head with capillary optrode using a fluorescent, sensitive and light-scattering layer [66]; **b)** Head with capillary optrode using 2 fluorescent, sensitive and light-scattering layers [67].

The application of fluorescent layers in optrodes combined with the use of optical filters can eliminate the influence of the light source on the photodetector. The light-scattering layer can be used to couple light into the capillary. The combination of light-scattering and fluorescent layers means that a sensor can be constructed without optical fibers. The fluorescent and sensitive layers have fluorescent properties only after they react with the substance being tested. A sensor head using a carbon-dioxide sensitive fluorescent and light-scattering layer is shown in Figure 9 [66].

The liquid monitoring procedure is simpler here than in the previously described designs because the requirement to insert the immersive liquid is eliminated. Light is coupled to the capillary walls by light scattering in the sensitive layer. The coupling efficiency can be increased by adding a reflector, but at the cost of a more complicated capillary positioning procedure. This sensor construction is suitable for integration. The electrical connections are far more flexible than are optical fibers, The construction presented on Figure 9a [66] was improved by depositing more than one fluorescent sensitive layer, as shown in Figure 9b, and measuring fluorescence lifetimes, [67]. The optrode presented there can be used for monitoring oxygen, nitrogen mixtures or air in liquid with applications the field of emergency medicine as well as for on-site environmental monitoring. A problem in practical applications lies in the sensitive layer’s stability in its normal environment that contains carbon dioxide. This dilemma has to be overcome in all sensors with sensitive layers [68].

A four-band capillary optical immunosensor for the simultaneous determination of mesotrione, hexaconazole, paraquat and diquat was developed by Mastichiadis *et al*. Four distinct bands (each corresponding to a different analyte) were created in the internal walls of a plastic capillary by immobilizing protein conjugates of the analytes. To perform the assay, the capillary was filled with a mixture of anti-analyte-specific antibodies together with a standard or sample containing the analyte(s). After a short incubation, a mixture of the appropriate second antibodies labeled with fluorescein was introduced into the capillary. To measure the fluorescence intensity bound onto each band, the capillary was scanned, perpendicularly to its axis, by a laser light beam. Part of the emitted photons were trapped into the capillary walls and waveguided to aphotomultiplier placed at the one end of the capillary. The analytical characteristics of the assays were comparable with those of the corresponding single-analyte fluoroimmunoassays performed in microtitration wells, proving the ability of the proposed immunosensor for reliable multianalyte determinations. The combination of low-cost disposable plastic capillary tubes with the low consumption of reagents, the short assay time, and the multianalyte feature of the proposed immunosensor indicated its potential for environmental analysis [69].

With the support of the EU-funded CLINICIP consortium mentioned in Section 1, capillary based carbon dioxide sensors were developed and tested, with the final aim of *p*CO_2_ monitoring in adipose tissue of critically ill patients [70]. The sensors were based on the measuring principle of phase fluorometry using a dual luminophore referencing scheme (DLR) to convert the CO_2_ dependent intensity signal into phase domain. The CO_2_ sensors were prepared by incorporating two appropriate luminophores and a phase transfer agent in a same hydrophobic polymer as matrix. A short-lifetime luminophore was used as pH indicator while the second inert luminophore was the long-lifetime dye. The capillary sensor and the planar sensor developed alongside were characterized and their construction and choice of materials optimized to achieve the best sensitivity and mechanical stability. The two glass capillary sections used in the measurement unit were each 2.8 cm long, 0.45 mm i.d and 1 mm o.d. The capillary preparation process included cleaning in acid solutions and thermal drying steps. Several polymers were examined as appropriate matrix for incorporation of two indicators. The largest phase shift up to 13° and 15° was observed for the best combination which was in detail examined in terms of sensitivity and stability. The sensors enabled the measurement of *p*CO_2_ in the range from 5 to 150 mmHg, with a resolution of 0.5 mmHg over the whole range and an accuracy of ±1 mmHg absolute or less than 7% of the read-out value. The planar sensors were characterized in dry gas and in solutions, while the capillary sensors were characterized in solutions.

Within the same CLINICIP consortium project an optical fiber sensor for measuring the pH in interstitial fluid was developed, based on microdialysis for extracting the sample from the subcutaneous adipose tissue. The interstitial fluid was drawn through a microfluidic circuit formed by a microdialysis catheter in series with a pH glass capillary. The pH indicator (phenol red) was covalently immobilized on the internal wall of the glass capillary. An optoelectronic unit that made use of LEDs and photodetectors was connected to the sensing capillary by means of optical fibers. A resolution of 0.03 pH units and an accuracy of 0.07 pH units were obtained [71]. Further investigations of researchers from the University of Technology of Graz and other members of the CLINICIP consortium, went in the direction of integrated fiber-optic hybrid sensors not based on FOCaps [72,73].

### 3.4. Optical Fiber Sensors Using Capillary Probes

**Figure 10.**
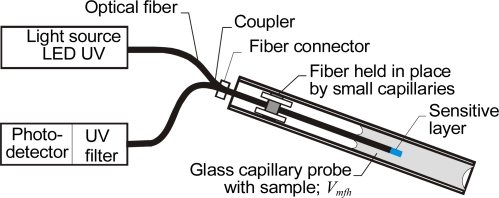
Optical fiber sensor using a capillary probe.

Optical fiber sensors with sensitive layers deposited on the fiber tip are well developed [74]. These sensors can operate in the reflection configuration where the fiber tip is in contact with the investigated medium [75]. This design may use single-mode fibers with circulators [76], multimode fibers with couplers, or large-core fibers (with core radius greater than 100 μm) with either custom-made directional couplers [77] or dividers that meet the TOSlink standard. Nanostructured sensitive layers are often deposited on the fiber tip [78], so the tip can be mechanically sensitive. The tip can then be inserted into the capillary or the glass part of a Pasteur pipette. One example of this design is an optical sensor of lead ions [79], in which the fiber and the capillary are positioned in the outer tube. The indicator is deposited on the tube walls where the introduced sample can dissolve it. Such construction enables relatively easy and repeated insertion of a bare fiber into the Pasteur pipette with the brackets fixed on the wider part of the pipette and on the outer tube. Keeping both ends of the capillary open enables pumping of the sample through capillary action, but assumes that the fiber tip is straight for a distance of a few centimeters, which is not always the case, as is known from fiber vibration sensors. A more precise insertion of the fiber into a capillary with the same geometry can be automated using an optical fiber splicer equipped with a monitoring camera. The fiber can also be positioned inside the capillary by means of a culvert created by a pair of capillaries whose outside diameters are smaller than the inner diameter of the sampling capillary, as illustrated in Figure 10.

In a device with this design, the distance between the sensitive layer and the fiber position can be of the order of a few millimeters. When a bare glass optical fiber is inserted into the glass capillary, the capillary action is forced right around the fiber, so this area fills even when basic capillary action of probe is not sufficient.

The capillary probe acts as a shield against outside light, because the capillary has a higher index of refraction than the examined liquid [80]. In that case the light reflection from the outer liquid meniscus is directed to the capillary walls. Both the inner diameter and the material of the small capillary sections used as culverts can be selected with a view to preventing the liquid from moving outside of the probe. The fiber connector makes it easy to disconnect the head for cleaning or replacement. Components recommended for this device are probe capillaries with an inner diameter of 700 μm (TSP700850), fibers with an outer diameter of about 300 μm (FVP300330370 with removed buffer in the optrode area) and small capillaries with an outer diameter of 150 μm (TSP050150 with thin layer of PMMA deposited on the inner walls), and SMA fiber connectors. Due to its simple operation and the fact that its head can be quickly replaced, the sensor in Figure 10 has good potential for use in point-of-care applications. This is investigated in [79] and [81].

### 3.5. Sensors with Chemically Preprocessed Samples

**Figure 11.**
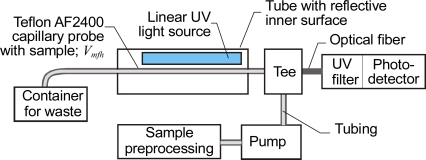
Capillary sensor scheme to monitor transfluent samples using fluorescence detection.

Sensors that work with chemically preprocessed samples often use fluorescence detection as it can be very selective. A capillary sensor scheme for monitoring transfluent fluorescent samples is shown in Figure 11.

A Teflon AF2400 tube is used here as the capillary probe. Teflon has an index of refraction slightly below 1.29 and a smooth surface which scatters light. These features enable it to side-couple light into the liquid in the tube where it can propagate. Miniaturized UV LEDs can be used as the light source for fluorescence investigation. The main drawback with this scheme lies in production of the necessary selective fluorescence in the presence of the investigated component, which must be a chemical reactant. To give two examples of research in this area: fluorescent sensors for saccharides have been developed based on the interaction between a boronic acid and a diol [82], and saccharide-selective systems have been developed for D-glucosamine, which is an amino sugar that stimulates collagen and cartilage production in the human body [83]. The problem is that some of the chemical reactions needed for fluorescence detection also produce gases, and this output complicates the optical detection of light propagated in the liquid waveguide. This was the experience of researchers working on sensors for the selective detection of NH_3_ ions in the range 35 nM–60 μM as presented in [84].

### 3.6. Capillary Sensors of Transparent Liquids with Light Switching by Local Sample Heating

**Figure 12.**
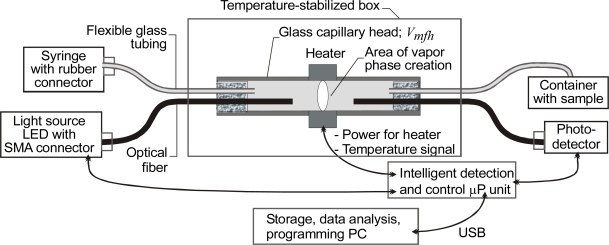
Fiber-optic capillary sensor of transparent liquids with local heating [87].

In this section, we review research carried out by the authors on capillary sensors using local heating of the transparent liquids. The selectivity of sensors can be achieved by physical processing of the sample in the capillary head, for example by heating [85–86]. In one sensor using that principle, the set of parameters from which the fingerprint of a liquid is selected was indirectly monitored by measurements of light propagation. To collect this information, the sample was subjected at specified times to local heating with a defined power level. Under certain conditions, such heating can result in the formation of a bubble of liquid vapor. After the heating stops, the bubble either becomes smaller or is completely absorbed by the liquid. The bubble itself forms a lens and by its presence and shape switches the light in the capillary, as illustrated in Figure 1. A fiber-optic capillary sensor using the described principle was proposed in [87]. The head of that sensor, with the optical fibers fixed in position inside the capillary head and flexible glass tubing used for sample injection, is presented in Figure 12.

In the setup shown in Figure 12, the optical fibers were positioned symmetrically in relation to the heater. The distance between the fibers was 15 mm; the distance between the glass tubes was 40 mm. Both the TSP 700850 capillaries used for the capillary head and the TSP 250350 used as the flexible glass tubes came from Polymicro Inc. The BFL22–200 optical fibers were from Thorlab. The inner diameter of TSP 700850 is 700 μm +/−10 μm and its outer diameter is 850μm +/− 20 μm, while for the BFL22–200 the silica core is 200 μm and the silica clad is 240 μm. The key to the repeatable operation of the sensor is the precise positioning in one plane of the BFL22–200 fibers and the TSP 250350 tubes. The heater, made by thick-film technology, had a width of 1.5 mm, and its heating power was 3 W. The temperature in the box where the head was placed was stabilized at 22 °C. This box was also used for removing vaporized liquid from the capillary as suggested in [86].

The signal received during and after sample heating depends on the geometry of the head, on the characteristics of the fibers and on the indexes of refraction of the capillaries as well as on the set of liquid parameters presented in Table 1 which includes data published in [88]. When the temperature of the liquid decreases, the vapor pressure also decreases and the bubble gets smaller. When the vapor pressure in the bubble is lower than the sum of the atmospheric pressure plus the pressure from surface tension, the bubble is absorbed. The atmospheric pressure is 1.013·10^5^ Pa, so it is expected that when heated rapidly to 200 °C, the isopropyl alcohol will shoot out from the capillary, while the ethylene glycol gas will expand more slowly. Calculating the amounts of vapor flashed from the components of a liquid requires a trial-and-error iterative solution. Such a calculation is commonly referred to as the equilibrium flash calculation. It involves solving the Rachford-Rice equation [89].

**Figure 13.**
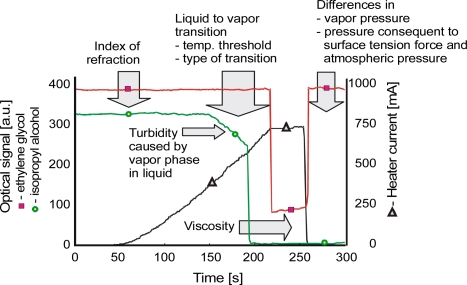
Signals registered from isopropyl alcohol and ethylene glycol liquid-to-vapor transition.

The constructed system was verified by using it to distinguish between two liquids: a 77.5% isopropyl alcohol-water solution and pure ethylene glycol. The optical signal transient characteristics for both samples are presented in Figure 13.

In the first 150 seconds of the experiment, a difference in light transmission between the capillaries filled with ethylene glycol and those filled with isopropyl alcohol was observed. The transmitted optical power was greater with ethylene glycol than with isopropyl alcohol because the refractive index of ethylene glycol is higher than that of isopropyl alcohol (Table 1). During the next 150–200 seconds, differences were seen in the threshold temperature for the formation of vapor bubbles. Longer times and higher temperatures were needed for ethylene glycol than for isopropyl alcohol, which is consistent with the difference in their boiling points as given in Table 1. With ethylene glycol, which has a high viscosity, the vapor bubbles are rimmed with thin layers of the liquid left on the inner capillary walls after the bubbles form. In effect, the transmission of light does not drop to zero, as it does in isopropyl alcohol whose viscosity is lower. On Figure 13, at the end of the measuring cycle, *i.e.*, after 250 seconds, the difference of signal levels due to different thermodynamic properties of vapor and the atmosphere is clearly visible.

It is worth noting that irrespective of the analyzed liquid, the signal waveforms of the consecutive measuring cycles have good reproducibility [87]. Thus, the characteristic features of the particular signal collected and analyzed using an ANN can serve to identify the type of liquid examined. In the ANN experiments, the characteristic points were taken to be: (a) the signal level at the beginning, (b) the signal magnitude just before boiling, (c) the time when the rate of the signal decrease begins to exceed 200 a.u./s, (d) the magnitude of the signal at its minimum value and (e) the magnitude of the signal at the cycle end. The neural network used was a multilayer perceptron with 5 inputs, 1 output and 2 hidden layers with the sigmoid transfer function in each neuron. The structure of the outputs was designed to make it simple to interpret the results. The output signal representation was 1-EG, 0-IPA. The ANN was taught on 26 cases. The learning error of the ANN for 1,000 iterations of the back propagation algorithm was 1.5% and the correlation of the training set was 0.9996. This means that the network parameters were well selected and confirms that the process of learning the desired features took place. The learning error value should be used as the criterion for accepting the accuracy of liquid classification. The liquid samples that give signals beyond the ranges defined as 0÷0.15 and 0.85÷1 may be considered to differ from the postulated kind. The percent of contribution from the inputs to the ANN output were: (a) 2%, (b) 2%, (c) 2%, (d) 35%, and (e) 59%. This proves that information on the index of refraction is of little importance when only one criterion is used.

**Figure 14.**
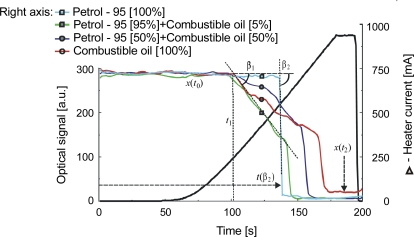
The vapor phase creation in selected fuel mixtures.

The bubbles in boiling liquid arise first on the surfaces of the container and the surfaces of impurities in the liquid. During heating, because the capillary is smooth inside, impurities can cause the formation of very small bubbles of vapor just before the boiling point is reached. This effect can be used to monitor liquid purity, which is an important goal in fuel quality assessment, and was therefore evaluated on two mixtures of Petrol 95 and combustible oil (95%–5% and 50%–50%). The signals received are presented in Figure 14. Fuels with similar refractive indexes were used, so the initial level of signal *x*(*t*_0_) is also similar. The speed of the liquid-to-gas transition can be defined by angle *β*_1_ (Figure 13). A higher value of *β*_1_ indicates a purer liquid. The time of boiling *t*(*β*_2_) can be found at the point where the optical signal rapidly decreases (*β*_2_ trend to 90°). In Figure 14 one can see that the *β*_1_ transition angle is equal to *β*_2_ in the case of pure petrol and that *β*_1_ decreases when there is more combustible oil in the mixture. The temperature of the boiling point also increases when the mixture contains higher levels of combustible oil. So, one can deduce that Petrol 95 is purer than combustible oil, a conclusion that reflects the fact that it is less refined and filtered during its production process.

## 4. Capillary Sensors for Point-of-Care Applications

Weigl *et al.* have presented a review of non- and minimally instrumented microfluidics-based diagnostic devices, making a case for disposable-only diagnostics and for micro total analysis systems (μTAS) [90]. The sensor market for point-of-care applications is expanding rapidly because it targets low-cost, fast self-diagnostics. The user population is growing, and self-diagnostics is becoming increasingly attractive as health-care costs continue to rise. Some of the best known diagnostic sensors were developed to examine organic secretions such as milk, vaginal fluids, sperm, lymph, saliva and blood. Organic secretions must be examined by disposable single-use microfluidic drawing devices with a permanent, reusable head, since it is difficult to wash or clean devices containing infected secretions. Minimizing the total sample volume needed for precise examination is one of the critical objectives in such applications. This can be achieved by using a capillary optrode to draw the sample into the microfluidic head.

**Figure 15.**
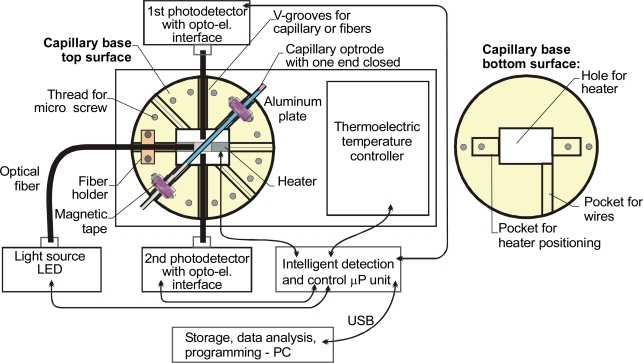
Microfluidic head with a replaceable capillary optrode.

The construction of a prototype testing head built with a replaceable capillary optrode built by the authors is shown in Figure 15. The base of this capillary was designed so that different size capillaries and fibers could be precisely positioned to test a wide range of organic secretions. The presented configuration uses two photodetectors. The position of the capillary in the base is controlled by the first photodetector and the measured signals presented on the following charts were registered from the second photodetector. The replaceable capillary optrode in this design is held in position by magnetic tape, the capillary base being made of magnetic steel. An aluminum plate serves to stabilize heat convection in the heated area, while the thermo-electric temperature controller sets a stabilized temperature for the experiments.

This setup uses TSP 700850 from Polymicro Inc., as well as special capillaries produced by the team of Prof. J. Dorosz at Bialystok Technical University [91] and BFL22-400 optical fibers from Thorlab. Its most important feature is that one end of the capillary is blocked after the sample is drawn. The capillary can be simply closed with modeling clay, which is inserted to a length of a few millimeters. This prevents any sample spilling and ensures a safe transfer from the place of draw to the point of examination. The sample must fill the capillary to about three-quarters of its length to ensure safe processing of the sample under heat.

The series of sensor evaluation runs described here used milk as the sample fluid [92]. Milk was chosen for this purpose because it is a very complex fluid and is relatively easy to obtain. Its optical properties provide the basis for many rapid, indirect methods of analysis such as proximate analysis by infrared absorbency or light scattering. These properties also determine the appearance of milk and milk products. For example, light scattering by fat globules and casein micelles causes milk to appear turbid and opaque. For this reason, milk intended for use in spectrometric and light scattering examination setups is diluted at a ratio of 1:10,000 although in current practice it is not always simple to obtain an accurate dilution repeatedly. A further complication is that fresh milk micelles have such a range of sizes that the light scattering setups need sources and detectors operating from λ < 300 nm to the visible light range [93]. Such instrumentation is relatively costly.

The total content of solids in milk can be estimated from its refractive index, which is between 1.3440 and 1.3485. Skimmed milk with a normal pH level behaves as a Newtonian fluid; the parameters influencing its viscosity are the concentration of solids, temperature and heat treatment [94]. Casein is a major contributor to the viscosity of skim milk. Most, but not all, of the casein proteins exist in a colloidal particle known as the casein micelle. Under normal conditions, casein micelles have a spherical shape, with diameters from 40 to 300 nm. The number of such particles in a milliliter of milk is in the range from 10^14^ to 10^16^, enough to cover a total surface of 5 × 10^4^ cm^2^/mL [95]. Changes in the physicochemical properties of the casein micelles can be induced by changes in pH, salt concentration, or temperature. Of interest are the time effects on these factors.

Non-pasteurized and non-sterilized milk ferments at room temperature. Lactic acid bacteria such Lactobacillus, Lactococcus or Leuconostoc convert milk monosaccharides (simple sugars, such as fructose) into lactic acid and energy, according to the following formula:

(4) $$C_{6}H_{12}O_{6}+\text{lactic acid bacteria}\to 2{\text{CH}}_{3}\text{CHOHCOOH}+22.5\;\text{kcal}$$

**Figure 16.**
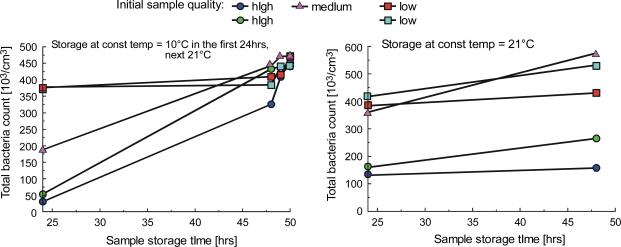
Total bacteria count of raw milk samples stored under different conditions.

The presence of lactic acid significantly lowers the pH level of milk. Low pH (pH of 4.6 at 20 °C) leads to casein coagulation, resulting in milk clot formation. The milk quality is determined by the level of bacteria present in it, but the exact limits vary from country to country. For our study we defined high quality milk as containing less than 50,000 bacteria per 1 cm^3^, the medium quality as containing up to 300,000 bacteria per 1 cm^3^ and beyond that level as low quality. The quantities of bacteria are not uniform in milk samples and can change over time at different rates, the milk quality remaining constant only during the first 24 hours of storage at 10 °C, as set forth in Figure 16.

**Figure 17.**
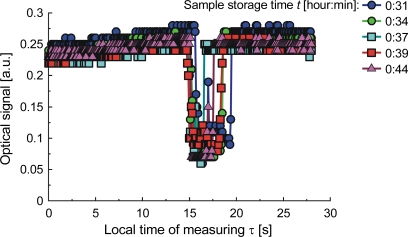
Signals from milk samples after storage of 31 to 44 minutes.

**Figure 18.**
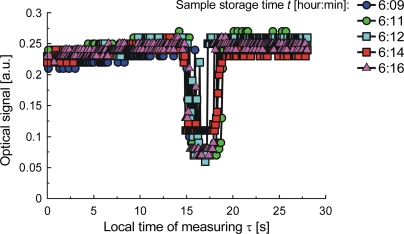
Signals from milk samples stored for about 6 hours.

**Figure 19.**
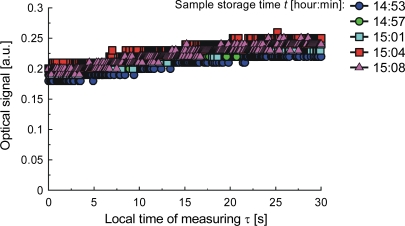
Signals from milk samples stored for about 15 hours.

To explore the influence of temperature on the structure of milk, we examined samples prepared from commercial milk powder and bottled water, stirred with a magnetic stirrer. The sample was stored at 20 °C until there were visible differences in the measured characteristics, which relate to the action of airborne bacteria. The samples were examined at temperatures up to a maximum of 90 °C, or until bubbles appeared. The appearance of a bubble is accompanied by a significant depression in the strength of the optical signal, which is not scattered. This happens when the milk is fresh. When the milk is spoiled by bacteria growth, a clot will form in the capillary upon local heating, and the signal depression will look different as illustrated by signals from samples stored for a time ranging from 31 to 44 minutes (Figure 17), for 6 hours (Figure 18) and for 15 hours (Figure 19). The differences of the signals are due to the increasing number of bacteria and can be calibrated in terms of the bacteria count [92]. The classification of samples by storage time was investigated with ANN. To create the sample model, we measured optical signal magnitude at time points of 0, 5, 12.5, and 30 seconds from the start of the measuring procedure. These points are labeled respectively as: *S*_1_(τ = 0 s), S_2_(τ = 5 s), *S*_3_(τ = 12.5 s), *S*_4_(τ = 30 s). Two other symbols used here are: τ*_hoff_*–time when the heater was turned off, and *S_stdmin_* – standard deviation of the signal amplitude at its minimum. The ANN output configuration was {1,0} for samples stored for less than 44 minutes and {0,1} for storage times greater than 6 hours. The correlation coefficient of learned ANN outputs with assumed outputs was 0.94 and the root mean square (RMS) error was 0.049 which means that the network can clearly distinguish the samples. The signals S_2_(τ = 5 s), *S*_3_(τ = 12.5 s) and τ*_hoff_* contain 70% of the information. The possibility of classification to reveal the presence of bacteria in the tested milk samples stored for times of up to 14 hours is not obvious from Figures 17 and 18, but with ANN can be done. When the storage time was extended to 15 hours, the correlation coefficient for the same model between the learned and the measured outputs was 0.98. As expected, this is a higher value than for the previous case, indicating that a longer time of sample storing simplifies the test conditions.

The interesting point here is that in this case the information is spread almost uniformly over the signal model with the exception of *S_stdmin_*, which means that parameter does not contribute significantly to the characterization. From the above experiments, it is clear that a replaceable capillary optrode works well and is capable of classifying milk with accuracy comparable to that of the fixed construction sensor shown in Figure 12.

## 5. Conclusions

Capillary sensors are highly promising for microfluidics applications that require a very low total sample volume. The chemically sensitive layers enable selective monitoring of liquids but have a limited lifetime. Equipped with local sample heating and information processing capability, capillary sensors merit further development work. They need no chemical reagents. The selectivity of tests incorporating the use of an ANN reaches far beyond what could be obtained with classical photonic sensors and confirms the potential of the proposed devices as useful tools for analyzing the composition of liquids, with the possibility of important practical applications in rapid diagnostics. The operation of capillary sensors is relatively simple, particularly when replaceable optrodes are used, making them well suited for applications in point-of-care systems. However, to be accepted for widespread consumer and industrial applications, they will need much more system integration, ideally leading to hand-held devices. This step will require embedding of light sources and detectors in a single plane, but in separated areas, which should be achievable using hybrid technologies.

## Figures and Tables

**Table 1.** Parameters of liquids: Water–DIW, Isopropyl alcohol –IPA, Ethylene glycol–EG.

**Parameter**	**Liquid**		
	**DIW**	**IPA (77.5%) + DIW (22.5%)**	**EG**
Index of refraction	1.333	1.376	1.432
Boiling point [°C]	100	82.3	197.3
Vapor pressure of liquid at 20 °C [Pa]	2.4·10^3^	4.4·10^3^	8
Vapor pressure of liquid at 100 °C [Pa]	1.0·10^5^	2.0·10^5^	1.4·10^3^
Vapor pressure of liquid at 200 °C [Pa]	1.8·10^6^	2.3·10^6^	1.1·10^5^
Viscosity [Pa·s]	0.89·10^3^	2.07·10^3^	16·10^3^
Surface tension [N/m]	0.0731	0.0228	0.0477
